# Evaluation of Natural Radioactivity and Radiological Hazards Associated With Earthen Building Materials Commonly Used in Bureti, Kericho County, Kenya

**DOI:** 10.1155/tswj/6861124

**Published:** 2026-03-10

**Authors:** Rotich Charles Kipngeno, Nadir Omar Hashim, Margaret Wairimu Chege

**Affiliations:** ^1^ Department of Physics, Kenyatta University, Nairobi, Kenya, ku.ac.ke

**Keywords:** Bureti, earthen building materials, exposure, health risks, radiation hazards

## Abstract

This study investigates the radioactivity due to naturally occurring radionuclides in earthen building materials commonly used in Bureti, Kenya. Building materials derived from the earth’s crust often contain naturally occurring radionuclides, which may pose radiological health risks. In regions such as Bureti, Kenya, where earthen materials are widely used for construction, assessing their radioactivity is essential for public safety. Thallium‐activated sodium iodide detector was used to measure the activity concentrations of ^40^K, ^226^Ra, and ^232^Th in the said materials and average values of 106 ± 61, 104 ± 8, and 82 ± 6 Bqkg^−^¹, respectively, which are about three times higher than global averages were recorded. Radiological hazard parameters including radium equivalent activity, absorbed dose rate, annual effective dose rate (AEDR_IN_), hazard indices, excess lifetime cancer risk (ELCR), annual gonadal effective dose (AGED), and activity utilization index (AUI) were calculated to evaluate potential exposure risks. Indoor absorbed dose rates (mean 270.76 ± 18.84 nGyh^−^¹) and AEDR_IN_ (mean 1.00 ± 0.07 mSv y^−^¹) exceeded international reference values, with 73.3% of samples surpassing the European Commission limit of 1 mSv y^−^¹. While external hazard indices (mean 0.83) were largely within safe limits, internal hazard (mean 1.10) and gamma indices (mean 1.11) exceeded permissible thresholds. ELCR (mean 3.49 × 10^−3^), AGED (mean 997.27 *μ*Sv y^−1^), and AUI (mean 2.04) values were significantly above global standards, indicating elevated long‐term health risks. The findings demonstrate that Bureti earthen building materials contain enhanced radionuclide concentrations, rendering them radiologically unsafe under international guidelines. Continuous monitoring and regulatory oversight are crucial to mitigate radiation exposure and safeguard residents’ health.

## 1. Introduction

Environmental pollution arising from ionizing radiations has been on the rise over the years [1]. This has resulted from any of the sources varying from natural, artificial, or occupational according to report from United Nations scientific committee on the effects of atomic radiation [2]. The largest percentage of radiation exposure received by the public amounting to 2.4 mSv y^−1^ (about 80%) of the total annual radiation dose is from natural sources [3]. About half of the radiation exposure from natural sources, that is, 42%, comes from inhalation of radon in indoor air while 58% is due to external exposure from (^226^Ra, ^232^Th, and ^40^K), cosmic radiation or through ingestion of food and water [4, 5].

Rocks and soil form a larger percentage of building materials in Bureti, and since they contain traces of ^232^Th and ^238^U as well as ^40^K, they can act as sources of the radiation exposure [6]. The levels of the radionuclides in these building materials majorly depend on the geological formation of the region and can as well vary from place to place. Higher radiation levels characterize igneous rocks such as granite, and on the other hand, sedimentary rocks are associated with lower levels of radiation [5]. Building materials emanating from the earth crust registered activity concentrations of about 500, 50, and 50 Bqkg^−1^ for ^40^K, ^226^Ra, and ^232^Th, respectively [7]. Natural radionuclides in these building materials are responsible for external as well as internal radiation exposures to the individuals residing in dwellings build from the same materials as most of them spend up to 80% of their time indoors [8, 9]. External exposure is due to gamma radiation arising from ^40^K, ^226^Ra, and ^232^Th together with their decay products, and on the other hand, internal radiation exposure results from the inhalation of radioactive radon isotopes (^222^Rn) and (^220^Rn) inert gases together with their short‐lived decay products [10, 11].

When houses are built from soil containing high concentrations of radionuclides, long‐term exposure to it can result in serious health issues such as thyroid nodules, prostate cancer, eye cataracts, and miscarriages in pregnant women [12, 13]. Our work reports the concentration levels due to natural radioactivity arising from earthen building materials commonly used in Bureti and the radiological health risks associated with them. This study was informed by the enhanced number of cancer cases recorded in the region as opposed to other subcounties of Kericho [14]. Furthermore, no radiological data on Bureti earthen building materials are available to guarantee the safety of using them for the construction of the dwellings. The results from this study will avail the information on the activity concentration as well as radiological hazards associated with radionuclides present in building materials. They will also provide basic guidelines on the use of these materials for building of houses by the locals as well as setting the standards for future references.

## 2. Materials and Methods

### 2.1. Study Area

**Figure 1:**
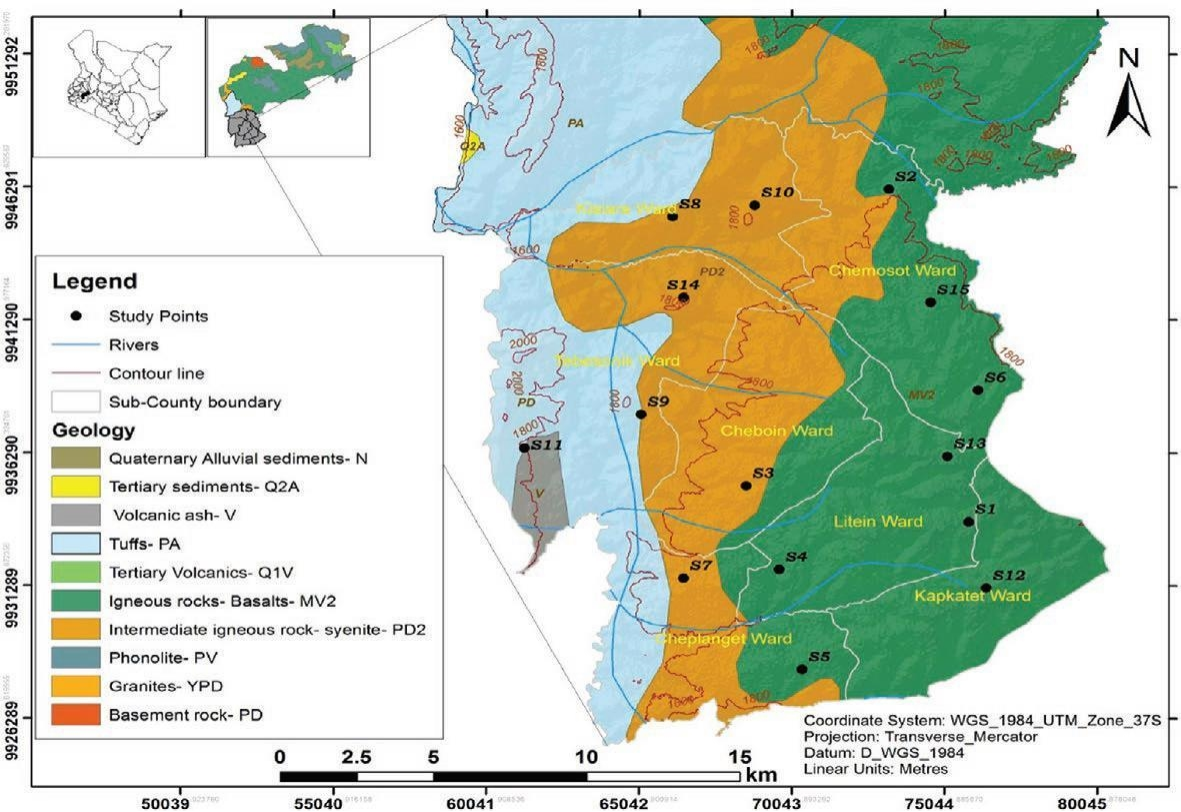
Map of Bureti region indicating sampling sites [15].

Bureti region, Kericho County lies in the Lake Victoria Basin on an altitude between 1950 and 2200 m above the sea level, longitude of 0°23 ^′^ S, 0°22 ^′^ N and latitude of 35°02 ^′^ W, 35°40 ^′^ E. As shown in Figure 1, it is characterized by volcanic as well as igneous rocks, metamorphic complexes, and soils which are known geological units for ^238^U, ^232^Th decay series, and ^40^K which are considered the primary sources of natural ionizing radiation [16–19]. It is also predominantly underlain by tertiary lavas (phonolites), intermediate basalt igneous rocks with the upper and the lower parts dominated by undifferentiated basement system rock (granites), volcanic ash admixture, and other prolific rocks [14].

### 2.2. Sample Collection and Preparation

Fifteen sampling sites in the study area, that is, Litein, Kapkatet, Mombwo, Chemoiben, Chebwagan, Cheborge, Tulwet, Kapkisiara, Cheplanget, Chemosot, Tebesonik, Kusumek, Roret, Kelunet, and Kaminjeiwet, were identified. Five mud walled houses were randomly selected from each of the sites, and a total of 75 mud samples of about 1 kg were cut from the walls of the houses and kept in well‐labeled polythene bags bearing the site numbers to avoid mixing of the samples during preparation. Five samples from every sampling site were mixed to form a sample, which is a representative of the larger sampling site [20]. Composite samples of about 300 g were packed in sealed plastic bags and labeled appropriately with sampling site name and number. These samples were then crushed into fine powder using a blender and allowed to pass through a 2‐mm sieve for homogenization.

The fine sediments were dried in an oven at a temperature of about 120°C to reduce moisture, and the sediments of about 200 g were stored in 250 mL polythene bags having the same geometry as that of the standard reference materials [21]. These containers were hermetically sealed and allowed to stay for 21 days before analysis to allow ^226^Ra and ^232^Th atoms to attain secular equilibrium with their daughters [22]. Spectral analysis of the earthen building materials was done at Kenyatta University physics laboratories using thallium‐activated sodium iodide detector interfaced with multichannel analyzer.

## 3. Experimental Procedure

### 3.1. Concentration Analysis of Radionuclides Using Gamma Ray Detector (NaI(Tl))

The gamma ray spectrometer used in this study consists of a 76 × 76 mm NaI(Tl) crystal detector and an Oxford PCA‐P card for the spectral data acquisition and analysis. The detector has energy resolution of 7.30% at 662 keV Cs −137 energy peak. Prior to spectral data acquisition, the device was calibrated using internationally accepted standard samples supplied and certified by international atomic (International Atomic Energy Agency). These samples are RGU‐1, RGTh‐1, and RGK‐1 and have the same geometry as the actual samples. Each sample was counted for a period of 30,000 s, and the background activity was determined by counting an inert sample that comprised distilled water in a polythene bag over the same duration. The primordial radionuclides ^226^Ra and ^232^Th are alpha emitters, and therefore, their activity concentrations cannot be measured using gamma ray detector, and therefore, their decay products that emit gamma rays were used.

The activity concentration of ^214^Bi (1765 keV) gamma ray emitter was used to determine the activity concentration of ^226^Ra while the energy peak of ^208^Tl (2615 keV) was used to determine the activity concentration of ^232^Th. The activity concentration of ^40^K was determined from its 1460 keV gamma energy peak. The region of interest was selected at the peak regions to obtain net counts of the full energy. The background counts were subtracted from the total counts to obtain residual counts for use in activity calculation.

The specific Activity A of the nuclides was determined using Equation (1) [23].

(1) A=Nbmγt,

where “*A*” is the specific activity of the gamma ray of interest, *N* is the net peak area of the gamma ray of interest, *b* is the emission probability of the gamma ray of interest, *m* is the mass of the sample in kilograms, *ɣ* is the detection efficiency of the gamma ray of interest, and *t* is the acquisition time in seconds.

### 3.2. Uncertainty‐Based Calibration of Radiation Detector Efficiency

Efficiency calibration of radiation detectors through uncertainty analysis is a precise metrological procedure that quantifies a detector’s performance in registering radiation events. This framework systematically propagates uncertainties from all contributing experimental variables such as background signal, measurement duration, and source activity. The outcome is a rigorously defined efficiency value accompanied by a quantified confidence level, ensuring both accuracy and reliability in measurement.

Uncertainty analysis was performed by propagating errors associated with spectral data acquired using NaI(Tl) detector. Uncertainties (±) were derived from Poisson counting statistics $\sigma =\pm \sqrt{N}$ and propagated through background subtraction, energy calibration, and detector resolution. Net beak areas were calculated using Equation (2).

(2) Ngross−Nbackground

With combined error expressed in Equation (3)

(3) σnet=±σ2gross+σ2background.

All reported values include propagated uncertainties and are expressed as *X* ± *σ*_*X*_, where *X* is the reported value, that is, net peak area, energy, efficiency, or activity, and *σ*_*X*_ is the uncertainty associated with that value, calculated and propagated from counting statistics, calibration, and detector resolution [24–27].

### 3.3. Radiation Hazards Estimation

#### 3.3.1. Radium Equivalent Activity

Radium equivalent activity is the weighted sum of activities of the three primordial radionuclides based on the estimation that 370 Bqkg^−1^ of ^226^Ra, 259 Bqkg^−1^ of ^232^Th, and 4810 Bqkg^−1^ of ^40^K yield the same gamma dose rates as expressed by Equation (4) [28, 29].

(4) Raeq=ARa+1.4290.0769 ATh+ AK,

where *A*_*R* *a*_, *A*_Th_, and *A*_*K*_ are the specific activity concentrations of ^226^Ra, ^232^Th, and ^40^K radionuclides, respectively, in becquerel per kilogram while 1.429 and 0.0769 are the conversion factors for ^232^Th and ^40^K, respectively.

#### 3.3.2. Radiation Absorbed Gamma Dose Rate (ADR)

The ADR (nGyh − 1)in air considered 1 m above the ground surface is a quantity which represents the specific energy per unit mass deposited in living matter by ionizing radiation. Residents normally get exposed to terrestrial gamma radiations when they stay both outside and inside the building, but this work focuses on indoor doses received by Bureti residents living in dwellings constructed from earthen building materials sourced locally. This quantity is dependent on the concentration of the radionuclides in the earthen building materials, and it was calculated using Equation (5) [28, 30].

(5) ADRINnGyh−1=0.921.10.08ARa+ATh+Ak,

where *A*_Ra_, *A*_Th_, and *A*_*K*_ are the specific activities of ^226^Ra, ^232^Th, and ^40^K, respectively.

#### 3.3.3. Indoor Annual Effective Absorbed Dose (AED)

Indoor AED is considered as the measure of biological effect due to ionizing radiation on humans inside a dwelling constructed from the soil. This was expressed by use of Equation (6) [4, 28, 31].

(6) AED=ADRnGyh×87600.60.7h××SvGy

where AED is the annual effective dose rate in millisieverts per year, ADR is the absorbed dose rate in nanogray per hour, 8760 is the time in hours for a whole year, 0.6 is the indoor occupancy factor (Kenyans spent 60% of their time indoors), and 0.7 Sv/Gy is the gamma dose conversion factor from hourly absorbed dose rate to annual effective dose rate [5, 7, 31].

#### 3.3.4. Gamma Index (*I*_*ɣ*_)

The gamma index is used for the assessment of the excess of gamma radiation originating from building materials. It was calculated using the following equation as proposed by European Commission [32].

(7) Hɣ=AK3000Bqkg−1+ARa300Bqkg−1+ATh200Bqkg−1,

where *A*_*R* *a*_, *A*_*k*_, and *A*_*T* *h*_ are the specific activities of ^226^Ra, ^40^K, and ^232^Th radionuclides, respectively.

#### 3.3.5. External Hazard Index (*H*_ex_)

It is a numerical value designed to evaluate the potential health risks associated with external gamma radiation emitted by natural radionuclides contained in building materials. It quantifies the indoor gamma radiation dose exposure due to ^226^Ra, ^232^Th, and ^40^K radionuclides. Based on the National Institutes of Health (NIH) recommendations, a *H*_*e* *x*_ value of less than 1 is considered acceptable indicating a low indoor external radiation hazard. This quantity was calculated by use of Equation (8) [12, 28, 33, 34].

(8) Hex=AK4810+ARa370+ATh259≤1,

where *A*_*R* *a*_, *A*_*k*_, and*A*_*T* *h*_ are the specific activities of ^226^Ra, ^40^K, and ^232^Th radionuclides, respectively.

#### 3.3.6. Internal Hazard Index (*H*_in_)

This is a measure used in assessing the potential health risk due to inhalation or ingestion of radon together with its short‐lived decay products which are also radioactive. It was determined from the activity concentrations of specific radionuclides contained in the air, that is, ^226^Ra, ^232^Th, and ^40^K, using Equation (9) [12, 28, 33, 34].

(9) Hin=AK4810+ARa185+ATh259≤1,

where *A*_*R* *a*_, *A*_*k*_, and*A*_*T* *h*_ are the specific activities of ^226^Ra, ^40^K, and ^232^Th radionuclides, respectively.

#### 3.3.7. Annual Gonadal Dose Equivalent (AGDE)

The AGDE is a hazard parameter used to determine the potential effects of the specific activities of ^226^Ra, ^232^Th, and ^40^K on some organs such as gonads, bone marrow, and bone surface cells containing cells that are capable of undergoing rapid division. An AGDE value greater than 300 *μ*Svy^−1^ can cause leukemia in victims [5]. The AGDE emanating from Bureti earthen building materials was calculated using Equation (10) [5, 35, 36].

(10) AGDE μSv y−1=3.094.180.314ARa+ATh+AK,

where *A*_*R* *a*_, *A*_*T* *h*_, and *A*_*K*_ are the activity concentrations of ^226^Ra, ^232^Th, and ^40^K, respectively, in becquerel per kilogram. The numbers 3.09, 4.18, and 0.314 are the respective conversion factors that transform the activity concentrations of ^226^Ra, ^232^Th, and ^40^K into total dose received by the organs of interest.

#### 3.3.8. Activity Utilization Index (AUI)

This is a radiological index used for assessing the suitability of construction materials, for public use by estimating potential gamma radiation exposure. AUI provides information on whether the materials used for construction of dwellings are safe or not and a value below 1 indicates low risk. Equation (9) was used to compute the AUI by considering the relative contributions of terrestrial radionuclides, that is, ^226^Ra, ^232^Th, and ^40^K, to the total gamma dose [37–39].

(11) AUIARa50fRa+ATh50fTh+AK500fk1Bqkg−1,

where*f*_Ra_, *f*_Th_, and *f*_*K*_ with values 0.462, 0.604, and 0.041, respectively, represent the share contributed by each radionuclide to the overall radiation dose.

#### 3.3.9. Excess Lifetime Cancer Risk (ELCR)

In the context of radioactivity, ELCR is considered as an estimated increase in the probability of a population developing cancer over a lifetime arising from the exposure to ionizing radiation. It quantifies the additional risk above the naturally occurring cancer rate. The ELCR per million persons per year was determined from the following Equation (12).

(12) ELCR=AEDE×DL×RF,

where DL is the duration of life (70 years) and RF is the fatal cancer risk which according to ICRP, 60 is 0.05 (Sv^−1^) [30, 40–43].

## 4. Results and Discussion

Bureti earthen building material samples recorded a mean activity concentration of 1061 ± 61 Bqkg^−1^ with a range of 536 ± 25–1626 ± 101 Bqkg^−1^, 104 ± 8 Bqkg^−1^ with a range of 59 ± 3–160 ± 12 Bqkg^−1^, and 82 ± 6 Bqkg^-1^ with a range of 54 ± 3–140 ± 10 Bqkg ^−1^ for ^40^K, ^226^Ra, and ^232^Th, respectively, as shown in Table 1. The average activity concentrations of ^40^K, ^226^Ra, and ^232^Th in the samples was about three times higher than their world average values of 400, 35, and 30 Bqkg^−1^, respectively. All the samples analyzed recorded concentrations above 500, 50, and 50 Bqkg^−1^ for ^40^K, ^226^Ra, and ^232^Th, respectively, reported from building materials originating from the earth crust [44, 45]. These elevated activities could be linked to volcanic as well as igneous rocks, metamorphic complexes, and soils characterizing the region (Figure 1) which are known geological units for ^238^U, ^232^Th decay series, and ^40^K [15, 16 ,17, 18]. Bureti earthen building materials recorded average activity concentrations that are slightly higher than those recorded from clay bricks in the neighboring Bomet County for the same radionuclides [46]. On the other hand, they recorded lower average activities for both ^226^Ra and ^232^Th compared to 134 ± 10 and 431 ± 19 Bqkg^−1^ for the same radionuclides but higher than 247 ± 27 Bqkg^−1^ for ^40^K reported from similar materials in Mrima Hills in the coastal region of Kenya [47].

**Table 1:** Specific activity concentrations of ^40^K, ^226^Ra, and ^232^Th for Bureti earthen building materials.

S/no.	SITE	Specific activity concentrations (Bqkg^−1^)		
		^40^K	^226^Ra	^232^Th
S1	Chemoiben	1312 ± 77	128 ± 9	77 ± 5
S2	Chemosot	1317 ± 79	134 ± 10	80 ± 5
S3	Cheborge	629 ± 33	59 ± 3	54 ± 3
S4	Chebwagan	540 ± 28	74 ± 5	70 ± 4
S5	Cheplanget	822 ± 44	76 ± 5	67 ± 4
S6	Kusumek	1216 ± 72	119 ± 9	72 ± 4
S7	Mombwo	920 ± 53	85 ± 7	75 ± 5
S8	Roret	827 ± 45	94 ± 8	89 ± 7
S9	Tebesonik	1626 ± 101	84 ± 7	90 ± 7
S10	Tulwet	1254 ± 75	130 ± 10	140 ± 10
S11	Kelunet	867 ± 49	124 ± 10	85 ± 7
S12	Kapkatet	1329 ± 78	106 ± 8	76 ± 5
S13	Litein	1283 ± 74	113 ± 8	82 ± 6
S14	Kapkisiara	1450 ± 83	72 ± 4	103 ± 8
S15	Kaminjeiwet	536 ± 25	160 ± 12	74 ± 5
Average		1061 ± 61	104 ± 8	82 ± 6

**Figure 2:**
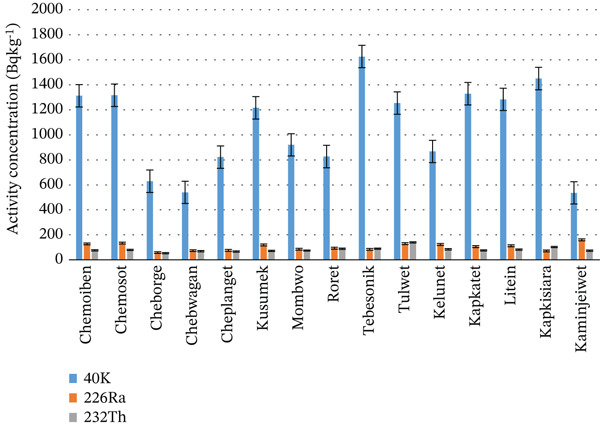
Activity concentrations of ^40^K, ^226^Ra, and ^232^Th for Bureti earthen building materials.

Neighboring sites of Chemoiben (S1), Chemosot (S2), Kusumek (S6), and Kapkatet (S12) as well as Litein (S13) reported enhanced activity concentrations for the three radionuclides, and this could be attributed to the same geological formations, and geographically, the sites are dominated by radioactive‐rich granite, phosphate, sandstone, and quartzite rocks and soil. Additionally, Tebesonik (S9), Tulwet (S10), and Kapkisiara (S14) as well reported raised activity concentrations for the same radionuclides. This could be as a result of deposition and accumulation of radioactive granite, phosphate, and quartzite soils washed by surface run off from the surrounding Tulwab‐mumek and Ngoina hills since the two sites are located on the lower terrain. Moreover, these are sites where marram for roads construction within and without Bureti is extracted resulting in environmental degradation. Though ^40^K compared to ^226^Ra and ^232^Th is naturally known to be abundant in soil and rocks, its dominance in the region as witnessed in Figure 2 can as well be linked to the use of phosphate fertilizers by the farmers to replenish the lost soil nutrients thus enriching the soil with potassium [5].

The radium equivalent activity registered a mean value of 302.77 ± 21.26 Bqkg^−1^ with a range of 184.54 ± 9.82–426.50 ± 30.06 Bqkg^−1^. As witnessed in Table 2, all the sites except (S10) representing 93.3% recorded radium equivalent activity values below permissible limit of 370 Bqkg^−1^ [48]. (S10) recorded an enhanced radium equivalent activity of 426.50 ± 30.06 Bqkg^−1^, and this is attributed to elevated values of the activities of both ^226^Ra and ^232^Th in the region. As shown in Table 2, all the samples recorded indoor absorbed dose rate values greater than the global mean value of 84 nGyh^−1^ [5] with a mean value of 270.76 ± 18.84 nGyh^−1^. This placed Bureti earthen building materials on the high side of the world average value of radiation dose rates in building materials. As shown in Table 2, the calculated values of indoor annual effective dose rate (AEDR_IN_) from terrestrial gamma radionuclides due to Bureti earthen building materials ranged from 0.60 ± 0.03 to 1.38 ± 0.1 mSv y^−1^ with a mean of 1.00 ± 0.07 mSv y^−1^. All the samples recorded AEDR_IN_ values higher than the recommended word average values of 0.40 and 0.07 mSv y^−1^ [5], and 73.33 % of the samples studied registered values greater than the European Commission limit of 1 mSv y^−1^ [30]. Based on the said limits, these building materials must be used with caution to mitigate radiation exposure to residents.

**Table 2:** Radium equivalent activity (Ra_eq_), outdoor and indoor absorbed gamma dose rates (*D*_*R*_), and indoor annual effective dose (AED) due to earthen building materials in Bureti.

S/no.	Site (S)	*R* *a*_*e* *q*_ (Bqkg^−1^)	AED_IN_(nGyh^−1^)	AED_IN_(mSv y^−1^)	AED_IN_(*S* *v* *y* ^−1^) × 10^−3^
S1	Chemoiben	338.93 ± 22.07	307.42 ± 19.94	1.13 ± 0.07	1.13 ± 0.07
2S	Chemosot	349.60 ± 23.22	316.64 ± 21.02	1.16 ± 0.08	1.16 ± 0.08
S3	Cheborge	184.54 ± 9.82	164.10 ± 8.70	0.60 ± 0.03	0.60 ± 0.03
S4	Chebwagan	215.56 ± 12.87	188.28 ± 11.24	0.69 ± 0.04	0.69 ± 0.04
S5	Cheplanget	234.95 ± 14.10	209.38 ± 12.52	0.77 ± 0.05	0.77 ± 0.05
S6	Kusumek	315.40 ± 20.25	285.96 ± 18.44	1.05 ± 0.07	1.05 ± 0.07
S7	Mombwo	262.92 ± 18.22	234.30 ± 16.18	0.86 ± 0.06	0.86 ± 0.06
S8	Roret	284.78 ± 21.46	250.54 ± 18.66	0.92 ± 0.07	0.92 ± 0.07
S9	Tebesonik	337.65 ± 24.77	306.36 ± 22.22	1.12 ± 0.08	1.12 ± 0.08
S10	Tulwet	426.50 ± 30.06	373.92 ± 26.20	1.38 ± 0.10	1.38 ± 0.10
S11	Kelunet	312.14 ± 23.77	276.94 ± 20.82	1.02 ± 0.08	1.02 ± 0.08
S12	Kapkatet	316.80 ± 21.14	287.44 ± 19.10	1.06 ± 0.07	1.06 ± 0.07
S13	Litein	328.84 ± 22.26	296.80 ± 19.88	1.09 ± 0.07	1.09 ± 0.07
S14	Kapkisiara	330.69 ± 21.81	295.54 ± 19.12	1.08 ± 0.07	1.08 ± 0.07
S15	Kaminjeiwet	306.96 ± 21.07	271.48 ± 18.54	1.00 ± 0.06	1.00 ± 0.06
Average		302.77 ± 21.26	270.76 ± 18.84	1.00 ± 0.07	1.00 ± 0.07

Analysis of the data presented in Table 3 reveals that more than 90% of the studied samples exhibited external hazard index values below the recommended safety threshold of 1 mSv y^−^¹, indicating minimal risk from external radiation exposure. In contrast, approximately 73.3% of the same samples recorded internal hazard and gamma index values exceeding the permissible limit, thereby highlighting a considerable potential risk associated with internal exposure pathways and elevated gamma radiation levels. These findings suggest that while external hazards remain largely within acceptable limits, internal radiological risks warrant closer scrutiny and mitigation. On the other hand, the hazard parameters such as ELCR and annual gonadal effective dose (AGED) were also determined and the calculated ELCR values, ranging between 2.10 × 10^−3^ and 4.83 × 10^−3^ with a mean of 3.49 × 10^−3^ as shown in Table 3, substantially exceed the global reference limit of 0.29 × 10^−3^ [5, 49, 50]. This finding highlights that the studied population is subjected to radiation exposure levels above internationally accepted safety standards, thereby indicating a potential long‐term health risk, particularly an elevated probability of cancer development. Similarly, AGED values ranged from 605.54 to 1380.66 *μ*Sv y^−1^ with an average of 997.27 *μ*Sv y^−1^, measuring above the world average value of 300 *μ*Sv y^−1^ [51]. Consequently, the presence of these materials in construction heightened long‐term health risks to sensitive organs, particularly the gonads, among individuals residing in the affected buildings. Likewise, the measured AUI values for Bureti earthen building materials with mean of 2.04 and a range of between 1.25 and 3.10 substantially exceed the global permissible limit of 1, thereby indicating that these materials are radiologically unsafe and unsuitable for use in construction applications under international standards.

**Table 3:** The associated radiological risk parameters in studied samples.

S/no.	Site (S)	Gamma index	External hazard index	Internal hazard index	Activity utilization index	Annual gonadal dose equivalent (*μ*Sv y^−1^)	Excess lifetime cancer *r* *i* *s* *k* × 10^−3^
S1	Chemoiben	1.26	0.92	1.26	2.22	1129.35	4.00
2S	Chemosot	1.29	0.94	1.30	2.31	1162.01	4.06
S3	Cheborge	0.68	0.50	0.66	1.25	605.54	2.10
S4	Chebwagan	0.78	0.58	0.78	1.57	690.82	2.42
S5	Cheplanget	0.86	0.63	0.84	1.58	773.01	2.70
S6	Kusumek	1.17	0.85	1.17	2.07	1050.49	3.68
S7	Mombwo	0.96	0.71	0.94	1.76	865.03	3.01
S8	Roret	1.03	0.80	1.02	2.01	922.16	3.22
S9	Tebesonik	1.27	0.91	1.14	1.99	1146.32	3.92
S10	Tulwet	1.55	1.15	1.50	3.10	1380.66	4.83
S11	Kelunet	1.13	0.84	1.17	2.24	1010.70	3.57
S12	Kapkatet	1.18	0.86	1.13	2.00	1062.53	3.71
S13	Litein	1.21	0.89	1.19	2.14	1094.80	3.82
S14	Kapkisiara	1.24	0.88	1.09	2.03	1108.32	3.78
S15	Kaminjeiwet	1.07	0.82	1.25	2.42	972.02	3.50
Average		1.11	0.83	1.10	2.04	997.27	3.49

The results of the present work were benchmarked against findings on building materials reported in different parts of Kenya and across the globe, as outlined in Table 4 [47, 52–60].

**Table 4:** The mean natural radioactivity concentrations in Bureti earthen construction materials compared with those from other parts of Kenya as well as other regions of the world.

S/no.	Country	Region	^40^K (Bqkg^−1^)	^226^Ra (Bqkg^−1^)	^232^Th (Bqkg^−1^)	Reference (s)
1	Kenya	Bureti	1061 ± 61	104 ± 8	82 ± 6	Present study
2	Kenya	Homa Hills	894	129	399	[52]
3	Kenya	Mrima Hills	249	134	421	[47]
4	Kenya	Baba Dogo, Nairobi	831 ± 42	378 ± 19	290 ± 15	[53]
5	South Africa	KwaZulu‐Natal	123 ± 0.83	156.89 ± 0.81	234.96 ± 1.15	[54]
6	Algeria	Northern Region	675.0 ± 4.0	65 ± 7.0	51.0 ± 5.0	[55]
7	Bangladesh	Dhaka city region	292.3 ± 43.7	29.5 ± 6.3	52.5 ± 12.2	[56]
8	China	North west region	713.0 ± 8.2	58.6 ± 4.7	50.4 ± 3.5	[57]
9	Greece	Thessaloniki area	710.0 ± 165	35.0 ± 11.0	45.0 ± 15.0	[58]
10	Malaysia	Peninsular region	7541 ± 272	241.0 ± 3.0	51.0 ± 4.0	[59]
11	Egypt	Cairo and its suburbs	227.0	24.5	24.4	[60]

## 5. Conclusion and Recommendation

This research provides a comprehensive assessment of natural radioactivity and associated radiological risks in earthen construction materials widely utilized in Bureti, Kericho County, Kenya. The radiological assessment of these materials reveals significantly elevated activity concentrations of ^40^K, ^226^Ra, and ^232^Th compared to global averages, with values approximately three times higher than international reference levels. These enhanced activities are strongly linked to the region’s geological formations volcanic, igneous, and metamorphic complexes as well as anthropogenic factors such as phosphate fertilizer use and soil deposition from surrounding terrains. While the radium equivalent activity for most sites remained below the permissible limit of 370 Bqkg−¹, one site (S10) exceeded this threshold, reflecting localized enrichment of radionuclides and buildings in this site should have adequate ventilation to minimize indoor radiation exposure.

More critically, the absorbed dose rates and annual effective dose rates (AEDR_IN_) consistently surpassed global mean values, with over 73% of samples exceeding the European Commission’s recommended limit of 1 mSv y−¹. This underscores a substantial radiological burden associated with indoor exposure. Hazard indices further highlight the risks: Although external hazard values were largely within safe limits, internal hazard and gamma indices exceeded permissible thresholds in the majority of samples. The calculated ELCR and AGED values were markedly higher than global reference levels, pointing to long‐term health risks, particularly increased cancer probability and potential harm to sensitive organs such as the gonads. Moreover, the AUI values, averaging above 2, clearly indicate that these materials are unsuitable for construction under international safety standards. Considering the studied radiological parameters, over 80% of them categorized Bureti earthen building materials as unsuitable for unrestricted use in construction since they can pose elevated long‐term health risks.

The research is not without constraints because its relatively small and less diverse sample may not fully capture the range of building materials used across the region. In addition, local geological variations that were not comprehensively addressed could influence the outcomes. The results also represent conditions at a single point in time, leaving out possible shifts in radioactivity levels driven by environmental or industrial changes. Even with these limitations, the study makes a significant contribution to understanding natural radioactivity in construction materials. By presenting detailed regional data, it highlights implications for building practices and offers insights that enrich broader discussions on radiological safety. It also sheds light on the role of local geology in shaping material radioactivity. These findings carry practical relevance for builders, architects, and policymakers in Kenya particularly in Kericho and comparable areas. They can serve as a foundation for developing safe use guidelines for construction materials and support public health strategies aimed at minimizing exposure to radiation from such sources.

Upcoming research ought to prioritize larger and more varied sample collections, encompassing different types of construction materials and wider geographic regions. Extended monitoring over time would help track shifts in radioactivity levels and measure the success of protective interventions. Exploring substitute materials and safer building techniques that reduce radiation exposure can further strengthen structural safety and promote environmental well‐being.

## Funding

No funding was received for this manuscript.

## Conflicts of Interest

The authors declare no conflicts of interest.

## Data Availability

The data used in this study are available from the corresponding author upon request.
